# Self-efficacy and quality of life mediate self-reported mental health outcomes in visual snow syndrome

**DOI:** 10.1038/s41598-026-36347-y

**Published:** 2026-02-03

**Authors:** Qing Huang, Xuanyue Yu, Haojie Gao, Xiao-xiao Lin, Jing Wang, Ping Su, Xiaofeng Li, Dong Chen, Dirk M. Hermann, Yi Liu

**Affiliations:** 1 Department of Neurology, Central Hospital of Dalian University of Technology, Dalian, 116033 China; 2 State Key Laboratory of Cognitive Science and Mental Health, Institute of Psychology, Chinese Academy of Sciences, Beijing, 100101 China; 3 Department of Psychology, University of Chinese Academy of Sciences, Beijing, 100049 China; 4 Peking University Sixth Hospital, Peking University Institute of Mental Health, NHC Key Laboratory of Mental Health (Peking University), National Clinical Research Center for Mental Disorders (Peking University Sixth Hospital), Beijing, 100191 China; 5 Department of Neurology, Center for Membrane Receptor and Brain Medicine, the Fourth Affiliated Hospital of School of Medicine, and International School of Medicine, International Institutes of Medicine , Zhejiang University, Yiwu, 322000 China; 6 Tongyang Municipal Engineering Co., Ltd, Dalian, 116025 China; 7 Department of Neurosurgery, Dalian University of Technology Affiliated Central Hospital, Dalian, 116033 China; 8 Institute of Cardio-cerebrovascular Medicine, Dalian University of Technology, Dalian, 116033 China; 9 Department of Neurology, University Hospital Essen, University of Duisburg-Essen, Essen, Germany

**Keywords:** Visual snow syndrome, Self-efficacy, Quality of life, Depression, Suicidal ideation, Psychology, Human behaviour

## Abstract

Visual snow syndrome (VSS) is a chronic neurological disorder associated with impaired mental health. While self-efficacy and quality of life (QOL) are known to influence mental health outcomes (depression and suicidal ideation) in clinical populations, their roles in VSS remain unexplored. This study aimed to examine the associations among VSS, self-efficacy, QOL, mental health outcomes and the potential serial mediation roles of self-efficacy and QOL. A cross-sectional study compared 64 VSS patients and 67 healthy controls matched with age, sex and education level. Participants completed validated questionnaires assessing self-efficacy (GSES), QOL (WHOQOL-BREF), depression (CES-D), and suicidal ideation (BSSI, first five items). Analyses included group comparisons, correlation analyses to examine variable relationships, multimodel linear regression and serial mediation modeling to test the hypothesized sequential pathway from VSS through self-efficacy and quality of life to mental health outcomes. Compared with controls, VSS patients demonstrated significantly lower self-efficacy (VSS: 23.6 ± 6.2; Controls: 30.6 ± 6.0; *p* < 0.001) and QOL (VSS: 62.5 ± 9.5; Controls: 73.6 ± 8.8; *p* < 0.001), alongside elevated depression (median [IQR]: VSS: 28 [21,34]; Controls: 11 [7, 15]; *p* < 0.001) and suicidal ideation (VSS: 6 [5, 7]; Controls: 5 [5,6]; *p* < 0.01). Serial mediation analysis revealed that the effects of VSS on depression and suicidal ideation were mediated through self-efficacy and QOL sequentially. The total indirect effect for depression was 7.73 (95% CI [5.64–9.85]), with QOL accounting for 49.09% of the total effect. For suicidal ideation, the total indirect effect was 0.80 (95% CI [0.39–1.22]). Lower self-efficacy and QOL appear to serially mediate the associations between VSS and mental health impairments. These preliminary, cross-sectional findings indicate that self-efficacy and QOL may serve as modifiable intervention targets that mediate or moderate the risk of depression and suicidal ideation in individuals with VSS. Clinicians should prioritize routine assessments of these factors to guide early intervention strategies, although longitudinal studies are needed to confirm these causal pathways.

## Introduction

Visual snow syndrome (VSS) is a chronic neurological disorder characterized by persistent visual disturbances resembling television static, accompanied by palinopsia, photophobia, and enhanced entoptic phenomena^1^. With a lifetime prevalence of 2.2%^2^, VSS represents a significant public health burden. Cross-sectional studies report that VSS patients exhibit significantly elevated rates of depression (25% reporting severe symptoms), anxiety, depersonalization, and sleep disturbances compared to healthy populations^3,4^.Although direct investigations of suicidal ideation in VSS remain limited, both visual impairment and chronic neurological conditions independently elevate suicide risk^5,6^. However, the psychological mechanisms linking visual symptoms to mental health deterioration remain poorly characterized, limiting the development of targeted interventions.

Previous research examining psychological mediators in VSS has been limited to direct pathways between visual symptoms and depressive outcomes^7,8^, failing to identify modifiable intervention targets that could guide clinical practice. Self-efficacy—defined as an individual’s belief in their capacity to execute behaviors necessary to produce specific performance outcomes^9^—has demonstrated robust associations with mental health outcomes across diverse chronic disease populations^10,11^. Critically, self-efficacy operates through its influence on QOL, defined by the World Health Organization as an individual’s perception of their position in life encompassing physical, psychological, social, and environmental well-being^12,13^. Theoretical frameworks postulate that self-efficacy enhances adaptive coping strategies, thereby improving QOL, which subsequently buffers against psychological distress^14,15^. Empirical evidence from chronic disease research supports this sequential pathway: self-efficacy predicts QOL improvements, which in turn reduce depressive symptoms and suicidal risk^16–18^ .

This serial model (VSS → self-efficacy → QOL → mental health outcomes) is theoretically more appropriate than alternative configurations because: (1) VSS diagnosis represents a stable clinical characteristic temporally preceding adaptive psychological processes, (2) self-efficacy as a belief system logically precedes its functional consequences on daily well-being (QOL), and (3) QOL impairments serve as proximal risk factors for subsequent mental health deterioration, as supported by intervention studies showing that enhancing self-efficacy improves QOL and thereby reduces psychological distress^19–21^.

Addressing these critical gaps in the VSS literature, the present study employed serial mediation analysis to examine whether self-efficacy and QOL sequentially mediate the relationship between VSS diagnosis and mental health outcomes of depression and suicidal ideation. We hypothesized that: (H1) VSS patients would exhibit significantly lower self-efficacy and QOL compared to healthy controls; (H2) VSS diagnosis would be associated with elevated depression and suicidal ideation; and (H3) the association between VSS and mental health outcomes would be serially mediated by reduced self-efficacy followed by impaired QOL. Confirmation of this theoretically grounded pathway would identify specific, modifiable intervention targets to mitigate mental health burden in this vulnerable population.

## Method

### Participants and procedures

Data collection occurred between December 2024 and January 2025. One hundred thirty-one participants were enrolled in this study, including 64 patients diagnosed with VSS (male: 51.6%, M = 29.39, SD = 6.52) and 67 healthy controls (male: 47.8%, M = 30.12, SD = 6.23). With the assistance of experienced therapists, 64 VSS patients from across China were recruited via a nation-wide patient group and provided with electronic questionnaires via the Questionnaire Star platform through QR codes. Participants in the healthy control group were recruited both online and via in-person word-of-mouth referrals, with the identical electronic questionnaires mentioned above.

Inclusion criteria for the VSS group were: (a) meeting the full diagnostic criteria for VSS^1^, (b) ability to provide informed consent and complete the online questionnaires. Exclusion criteria included: (a) presence of significant cognitive impairment, (b) Coexisting severe ophthalmic diseases (e.g., glaucoma, retinal detachment) or neurological disorders (e.g., epilepsy, stroke). Key demographic variables, including age, sex, and education level, were matched for VSS and healthy control subjects., with no statistically significant differences (*p* > 0.05) between these two groups. Healthy participants were screened in the structured questionnaires to confirm the absence of ophthalmological / neurological / psychiatric disorders or regular medication use.

The study was conducted in strict adherence to ethical guidelines. All research procedures were reviewed and approved by the Ethics Committee of Central Hospital of Dalian University of Technology (Approval No: YN2025-234-01). Informed consent was obtained from all participants before data collection, with assurances of data anonymity and confidentiality.

### Study variables

The General Self-Efficacy Scale (GSES; Schwarzer, 1995) is a 10-item self-report scale that assesses the self-efficacy displayed by individuals in dealing with various types of challenges. The Chinese version of this scale used in this study has been validated and widely used in Chinese populations^22^. The scale had excellent internal consistency (Cronbach’s *α* = 0.906) in our sample.

The World Health Organization Quality of Life Brief Version Scale (WHOQOL-BREF; WHO, 1998) is a self-report scale with 26 items designed to assess the quality of an individual’s existence in four domains: physical health, mental health, social relationships, and the environment. The questionnaire was scored via a percentage system in this study. The Chinese version of this scale used in this study has been validated and is widely used in Chinese populations^23^. The scale had excellent internal consistency (Cronbach’s *α* = 0.911) in our sample.

The Center for Epidemiologic Studies Depression Scale (CES-D; Radloff, 1977) is a self-reported scale with 20 items that measures the level of depressive symptoms in individuals. The Chinese version of the CES-D used in this study has been validated and widely used in Chinese populations^24^. The scale had excellent internal consistency (Cronbach’s *α* = 0.928) in our sample.

The Beck Suicide Ideation Scale (SSI; Beck, 1979) is a self-report scale used to assess suicidal ideation. The Chinese version of the SSI used in this study has been validated and widely used in Chinese populations^25^. The present study used the first 5 questions of the scale to assess suicidal ideation, which is supported by prior evidence for its validity in measuring suicidal ideation^26^. The scale exhibited acceptable internal consistency, as evidenced by a Cronbach’s alpha coefficient (Cronbach’s α = 0.744) in the current sample.

### Statistical analysis

Descriptive statistics of sociodemographic characteristics (age, sex, education), self-efficacy, QOL, depression, and suicidal ideation scores are reported as percentages (%), means ± standard deviations (SD), or medians (interquartile ranges [IQR]), according to variable features. Quantitative data following a normal distribution were compared using *t*-tests and expressed as means ± standard deviations. Non-normally distributed data were compared using non-parametric rank-sum tests and expressed as medians (IQR). Categorical variables are expressed as counts and percentages and were compared using chi-square tests.

To inspect the correlations between the variables of interest, estimates of Pearson’s bivariate correlations between VSS, self-efficacy, QOL, depression, and suicidal ideation were computed. Variance Inflation Factor (VIF) and Tolerance values were calculated, with VIF < 5 and Tolerance > 0.2 considered indicators of no severe multicollinearity, ensuring the reliability and stability of subsequent regression results.

To explore the pathway relationships between VSS, self-efficacy, QOL, and mental health outcomes (depression and suicidal ideation), multimodel linear regression analyses were conducted. Two adjusted regression models were established: Model 1 was adjusted for age and sex, while Model 2 further incorporated education as a covariate. The analysis focused on quantifying specific path coefficients, including VSS to self-efficacy (Path a1), VSS to QOL (Path a2), self-efficacy to QOL (Path d), VSS to depression (Path c), self-efficacy to depression (Path b1), QOL to depression (Path b2), VSS to suicidal ideation (Path f), self-efficacy to suicidal ideation (Path e1), and QOL to suicidal ideation (Path e2).

Serial mediation models were constructed to examine the mediating roles of self-efficacy and QOL in the association between VSS and mental health outcomes. Mediating effect analysis was performed via Model 6 in the Process 4.1 program compiled by Hayes, with the mediating effect tested by repeated sampling 5000 times through the bootstrap method. The models respectively focused on the mediating effects on the two mental health outcomes (depression and suicidal ideation), following the sequential path framework of VSS → self-efficacy → QOL → mental health outcomes. For each model, coefficients, standard errors, and t-values were calculated for each path to verify significance, and model fit indices (*R²*, *F*-statistics) were reported to evaluate the validity of the mediation effects. All data analyses were performed using SPSS 23.0, with mediation analysis conducted via the Hayes PROCESS 4.1 macro^27^, and a two-sided *p* < 0.05 was considered statistically significant.

## Results

### Baseline characteristics of participants

Table 1 presents the baseline characteristics of participants. The VSS group (*n* = 64) and healthy control group (*n* = 67) were well-matched on age (VSS: 29 ± 7 years; Controls: 30 ± 6 years; *p* = 0.515), sex distribution (VSS: 51.6% male; Controls: 47.8% male; *p* = 0.664), and education level (*p* = 0.530).

**Table 1:** Baseline characteristics of participants in the VSS and healthy Controls.

Characteristic	Controls (*n* = 67)	VSS (*n* = 64)	*p*-value
Age, Mean ± SD	30 ± 6	29 ± 7	0.515
Sex, n (%)			0.664
Male	32 (47.8%)	33 (51.6%)	
Female	35 (52.2%)	31 (48.4%)	
Education, n (%)			0.530
High School or below	8 (11.9%)	9 (14.1%)	
Associate Degree	18 (26.9%)	21 (32.8%)	
Bachelor’s Degree	32 (47.8%)	30 (46.9%)	
Master’s Degree or above	9 (13.4%)	4 (6.3%)	
Self-efficacy	30.6 ± 6.0	23.6 ± 6.2	< 0.001
QOL	73.6 ± 8.8	62.5 ± 9.5	< 0.001
Depression	11(7, 15)	28(21, 34)	< 0.001
Suicidal ideation	5(5, 6)	6(5, 7)	< 0.01

Continuous variables are presented as mean ± standard deviation (SD) and were compared using independent *t*-tests. Categorical variables are presented as n (%) and were compared using Chi-square tests. This figure shows the baseline demographic and clinical characteristics of participants in the VSS group and the Healthy Controls group. Significant differences were found in Self-efficacy, QOL, Depression, and Suicidal ideation between the two groups. VSS = Visual Snow Syndrome; QOL = Quality of Life.VSS = Visual Snow Syndrome. QOL = Quality of Life.

Compared to healthy controls, VSS patients demonstrated significantly lower self-efficacy (VSS: 23.6 ± 6.2; Controls: 30.6 ± 6.0; p < 0.001) and QOL (VSS: 62.5 ± 9.5; Controls: 73.6 ± 8.8; p < 0.001). VSS patients also reported significantly higher levels of depression (median [IQR]: VSS: 28 [21, 34]; Controls: 11 [7, 15]; p < .001) and suicidal ideation (VSS: 6 [5, 7]; Controls: 5 [5, 6];* p* < 0.01). The median depression score of 28 in the VSS group substantially exceeds the CES-D clinical cutoff of ≥16 [28]—validated in Chinese populations [29,30]—indicating moderate-to-severe depressive symptomatology, while the median score of 11 in healthy controls falls below this threshold, reflecting no clinically significant depression. While the difference in suicidal ideation reached statistical significance, the absolute median difference of 1 point was small, suggesting modest clinical impact.

### Correlational analyses

Table 2 presents the Pearson correlation coefficients among VSS, self-efficacy, QOL, depression, and suicidal ideation. VSS was significantly negatively correlated with self-efficacy (*r* = -0.502, *p* < 0.001) and QOL (*r* = -0.522, *p* < 0.001), while positively correlated with depression (*r* = 0.675, *p* < 0.001) and suicidal ideation (*r* = 0.261, *p* < 0.01). Self-efficacy showed a strong positive correlation with QOL (*r* = 0.636, *p* < 0.001) and inverse correlations with depression (*r* = -0.690, *p* < 0.001) and suicidal ideation (*r* = -0.313, *p* < 0.01). Additionally, QOL was significantly negatively associated with depression (*r* = -0.719, *p* < 0.001) and suicidal ideation (*r* = -0.512, *p* < 0.01), whereas depression and suicidal ideation exhibited a positive correlation (*r* = 0.419, *p* < 0.001). All correlations were statistically significant at the two-sided *p* < 0.05 level, confirming robust linear associations among the variables.

**Table 2:** Pearson correlations among VSS, Self-Efficacy, QOL, Depression, and suicidal Ideation.

Variables	VSS	Self-efficacy	QOL	Depression	Suicidal ideation
VSS	1				
Self-efficacy	-0.502***	1			
QOL	-0.522***	0.636***	1		
Depression	0.675***	-0.690***	-0.719***	1	
Suicidal ideation	0.261**	-0.313***	-0.512**	0.419***	1

Correlations were calculated using bivariate Pearson correlation analysis. This figure presents the Pearson correlation analysis used to assess the relationships between VSS, Self-efficacy, QOL, Depression, and Suicidal ideation. The correlations were computed to examine both direct and indirect associations among these variables. Statistical significance for all correlations was determined using a *p*-value threshold of 0.001 for highly significant correlations, with the results presented in the table showing the strength and direction of these relationships. VSS = Visual Snow Syndrome; QOL = quality of life. Values represent Pearson correlation coefficients. Significance levels: *p < 0.05, **p < 0.01, ***p < 0.001.

### Multimodel linear regression analysis

Collinearity diagnosis indicated that the VIF for all predictor variables was < 5 and Tolerance > 0.2, confirming no severe multicollinearity. Table 3 presents the results of multimodel linear regression analyses examining the pathway relationships among VSS, self-efficacy, QOL, depression, and suicidal ideation. The Model 1 (age- and sex-adjusted) and Model 2 (adjusted for age, sex, and education) showed a similar pattern of associations across all pathways. Both models confirmed that VSS significantly negatively predicted self-efficacy (Path a1: *B* = -7.09, *p* < 0.001) and QOL (Path a2: *B* = -11.15, *p* < 0.001), while self-efficacy positively predicted QOL (Path d: *B* = 0.94, *p* < 0.001). Notably, VSS consistently retained a direct positive predictive effect on depression (Path c: *B* = 16.01 *p* < 0.001) and suicidal ideation (Path f: *B* = 0.75, *p* < 0.01) across both adjusted models. Additionally, self-efficacy negatively predicted depression (Path b1: *B* = -1.16, *p* < 0.001) and suicidal ideation (Path e2: *B* = -0.07, *p* < 0.01). QOL negatively predicted both depression (Path b2: *B* = -0.81, *p* < 0.001) and suicidal ideation (Path e2: *B* = -0.07, *p* < 0.01).

**Table 3:** Multimodel linear regression analysis: relationships between VSS, Self-Efficacy, QOL, and mental health outcomes (Depression/Suicidal Ideation).

	Path	Model 1		Model 2	
		B (95% CI)	*p*-value	B (95% CI)	*p*-value
	a₁	-7.05(-9.15~-4.94)	< 0.001	-7.09(-9.17~-5.01)	< 0.001
	a₂	-11.20(-14.35~-8.06)	< 0.001	-11.15(-14.24~-8.06)	< 0.001
	D	0.97(0.77 ~ 1.18)	< 0.001	0.94(0.74 ~ 1.15)	< 0.001
Depression	b₁	-1.15(-1.35~-0.94)	< 0.001	-1.16(-1.38 ~-0.95)	< 0.001
	b₂	-0.79(-0.92 ~-0.66)	< 0.001	-0.81(-0.95~-0.68)	< 0.001
	C	15.94(13.00 ~ 18.87)	< 0.001	16.01(13.04 ~ 18.97)	< 0.001
Suicidal ideation	e₁	-0.07 (-0.10 ~ -0.03)	< 0.001	-0.06 (-0.10 ~ -0.03)	0.001
	e₂	-0.07 (-0.10 ~ -0.05)	< 0.001	-0.07 (-0.09 ~ -0.05)	< 0.001
	f	0.79 (0.27 ~ 1.30)	0.003	0.75 (0.25 ~ 1.26)	0.004

Model 1: Age and sex adjusted; Model 2: Adjusted for age, sex and education; Path a1 = VSS → Self-efficacy; Path a2 = VSS → QOL; Path d = Self-efficacy → QOL; Path c = VSS → Depression; Path b1 = Self-efficacy → Depression; Path b2 = QOL → Depression; Path f = VSS → Suicidal ideation; Path e1 = Self-efficacy → Suicidal ideation; Path e2 = QOL → Suicidal ideation. This figure presents the linear regression analysis of the associations between VSS, Self-efficacy, QOL, and mental health outcomes (Depression and Suicidal Ideation). Two models were used: Model 1 adjusted for age and sex, and Model 2 adjusted for age, sex, and education. The analysis estimates the direct and indirect effects of each path, including the impact of VSS on Self-efficacy, QOL, Depression, and Suicidal Ideation, as well as the mediating effects of Self-efficacy and QOL. *P*-values indicate statistical significance for each path.VSS = Visual Snow Syndrome; QOL = quality of life.

### Exploratory serial mediation analyses

#### Serial model of depression

**Fig. 1:**
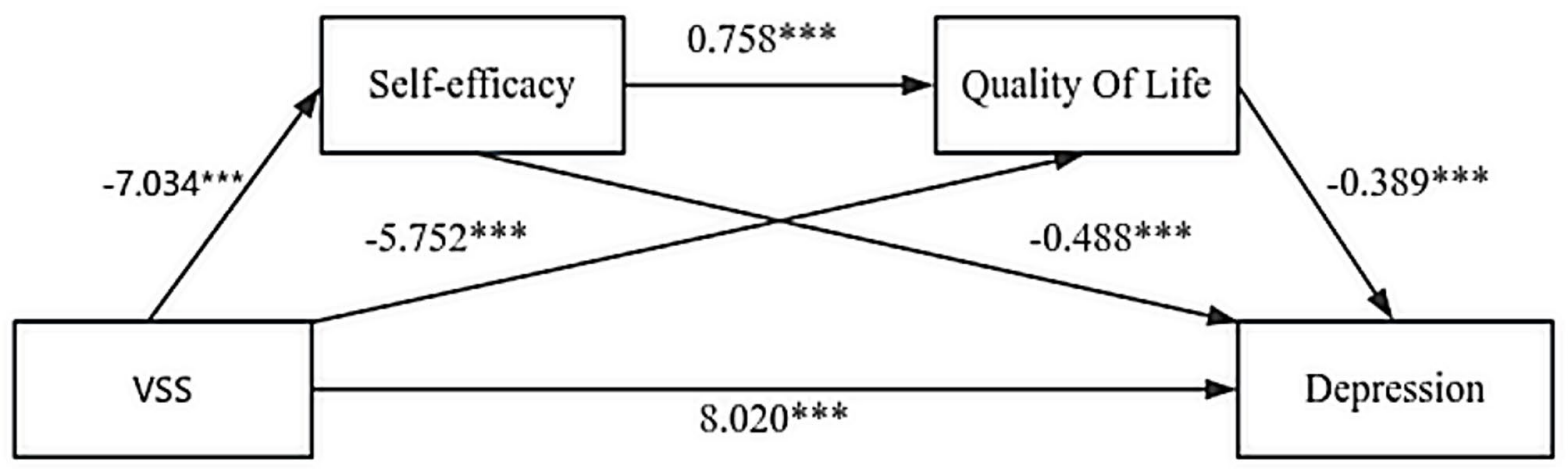
Serial mediation of self-efficacy and quality of life in the effect of Group on Depression. This figure presents the serial mediation model showing the effects of Group on Depression through Self-efficacy and QOL. The standardized regression coefficients (*B*) are shown for each path. Significant paths are marked with *** (*p* < 0.001), indicating strong relationships between the variables. *p < 0.05, **p < 0.01, ***p < 0.001.

The serial mediation model (VSS → self-efficacy → QOL → depression) was tested to explore the tenability of this specific theoretical pathway. The model results are presented in Table 4; Fig. 1. The model shows that the group significantly negatively affects self-efficacy, while both group and self-efficacy significantly impact QOL. Furthermore, group, self-efficacy, and QOL all significantly influence depression. Depression is indirectly affected by VSS through both self-efficacy and QOL.

**Table 4:** Relationships between quality of life-mediated self-efficacy and depression.

Antecedent	Self-efficacy				QOL				Depression			
	Coeff.	SE	*t*	*p*-value	Coeff.	SE	*t*	*p*-value	Coeff.	SE	*t*	*p*-value
VSS	-7.034	1.067	-6.591	< 0.001	-5.752	1.597	-3.603	< 0.001	8.020	1.403	5.715	< 0.001
Self-efficacy	-	-	-	-	0.758	0.114	6.651	< 0.001	− 0.488	0.111	-4.404	< 0.001
QOL	-	-	-	-	-	-	-	-	− 0.389	0.074	-5.248	< 0.001
Constant	37.646	1.676	22.464	< 0.001	56.174	4.806	11.690	< 0.001	46.924	5.788	8.108	< 0.001
	*R*^*2*^ = 0.252				*R*^*2*^ = 0.459				*R*^*2*^ = 0.688			
	*F* = 43.436, *p* < 0.001				*F* = 54.316, *p* < 0.001				*F* = 93.405, *p* < 0.001			

This figure presents the results of regression analysis to examine the relationships between QOL-mediated self-efficacy and Depression. The analysis involved multiple models: Self-efficacy and QOL as mediators in the relationship between group and Depression. The regression models also include the adjusted *R²* values and *F* statistics, with *p*-values indicating the statistical significance of each path. Coefficients (Coeff.) and standard errors (SE) are presented for each path. VSS = visual snow syndrome; QOL = quality of life; The mediating effect accounted for 49.9% of the total effect (Total effect = 15.754, Mediating effect = 7.734, Indirect/Total = 49.9%).

Percentile bootstrap analyses demonstrated significant effects in the serial mediation model. The total effect of VSS on depression was significant, with a point estimate of 15.754 (SE = 1.516, 95% CI [12.754, 18.754]). This total effect decomposes into a significant direct effect of VSS on depression (Effect = 8.020, SE = 1.403, 95% CI [5.243, 10.797]) and a significant total indirect effect (Effect = 7.734, SE = 1.058, 95% CI [5.638, 9.854]). The total indirect effect was further partitioned into three specific pathways: the indirect effect via self-efficacy alone (VSS → Self-efficacy → Depression) was 3.433, calculated as indirect effect 1 = − 7.034×(− 0.488); the indirect effect via QOL alone (VSS → QOL → Depression) was 2.238, calculated as indirect effect 2 = − 5.752×(− 0.389); and the indirect effect through the sequential pathway (VSS → Self-efficacy → QOL → Depression) was 2.063, calculated as indirect effect 3 = − 7.034 × 0.758×(− 0.389). The proportion of the total effect mediated by these pathways was (7.734/15.754) ×100% ≈ 49.09%, indicating that nearly half of the total effect of VSS on depression was explained by the serial mediation through self-efficacy and QOL.

#### Serial model of suicidal ideation

**Fig. 2:**
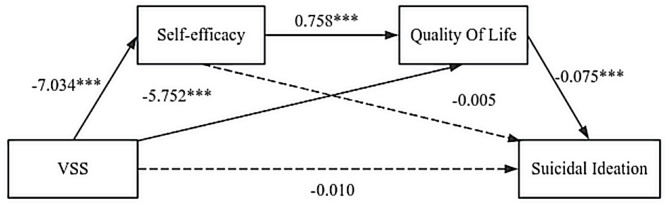
Serial mediation of self-efficacy and quality of life in the effect of Group on suicidal Ideation. This figure presents a serial mediation model illustrating the effects of Group on Suicidal Ideation through Self-efficacy and QOL. The unstandardized regression coefficients (*B*) are presented for each path. Significant paths are marked with *** (*p* < 0.001), indicating strong relationships between the variables. *p < 0.05, **p < 0.01, ***p < 0.001.

A key finding was the significant indirect association of VSS with suicidal ideation through self-efficacy and QOL, in the absence of a significant direct effect (see Table 5; Fig. 2). Percentile bootstrap analyses revealed a significant total effect (Effect = 0.794, SE = 0.258, 95% CI = 0.283 to 1.304), a nonsignificant direct effect (Effect = − 0.010, SE = 0.281, 95% CI = − 0.565 to 0.546), and a significant indirect effect (Effect = 0.803, SE = 0.211, 95% CI = 0.385 to 1.224). Notably, none of the 95% confidence intervals included zero, strongly supporting the significance of the mediating effect. Methodologically, a significant indirect effect does not require a significant total direct effect, and such a pattern can indicate that the influence of the independent variable is fully accounted for by the mediating mechanism. This result underscores the potential importance of the self-efficacy-QOL pathway in understanding suicidal ideation in VSS, even in the absence of a strong direct link between the diagnosis and ideation.

**Table 5:** Relationships between quality of life-mediated self-efficacy and suicidal ideation.

Antecedent	Self-efficacy				QOL				Suicidal ideation			
	Coeff.	SE	*t*	*p*-value	Coeff.	SE	*t*	*p*-value	Coeff.	SE	*t*	*p*-value
VSS	-7.034	1.067	-6.591	< 0.001	-5.752	1.597	-3.603	< 0.001	− 0.010	0.281	− 0.034	0.973
Self-efficacy	-	-	-	-	0.758	0.114	6.651	< 0.001	0.005	0.022	0.203	0.840
QOL	-	-	-	-	-	-	-	-	− 0.075	0.015	-5.085	< 0.001
Constant	37.646	1.676	22.464	< 0.001	56.174	4.806	11.690	< 0.001	11.015	1.158	9.510	< 0.001
	*R*^*2*^ = 0.252				*R*^*2*^ = 0.459				*R*^*2*^ = 0.688			
	*F* = 43.436, *p* < 0.001				*F* = 54.316, *p* < 0.001				*F* = 93.405, *p* < 0.001			

This figure presents the hierarchical regression analysis of the effects of Group, Self-efficacy, and Quality of Life (QOL) on Suicidal ideation. Three variables, Self-efficacy, QOL, and Group, are tested in relation to Suicidal ideation in the regression model. The *t*-values and *p*-values indicate the statistical significance of each path, with *p* < 0.001 showing strong relationships.VSS = visual snow syndrome; QOL = quality of life. Coefficients (Coeff.) and standard errors (SE) are presented for each path.

## Discussion

This study revealed that VSS patients exhibited widespread significantly lower self-efficacy and QOL scores, along with higher levels of depressive symptoms and suicidal ideation compared with healthy controls at baseline: significantly lower self-efficacy and QOL scores, coupled with elevated levels of depressive symptoms and suicidal ideation (*p* < 0.01). Serial mediation analysis further elucidated the pathways among variables. The effect of VSS on depression demonstrated partial mediation, operating through both a significant direct effect (*p* < 0.01) and an indirect pathway via the sequential chain of “diminished self-efficacy → compromised QOL.” In contrast, VSS showed no significant direct effect on suicidal ideation (*p* = 0.973), with the relationship being fully mediated through the same sequential pathway. The novelty of this study lies in documenting the elevated prevalence of suicidal ideation among VSS patients—a finding not systematically reported in prior VSS literature. Moreover, this represents the first demonstration that self-efficacy and QOL mediate the relationship between VSS and mental health outcomes, independent of age, sex, and educational attainment. While research in other disease populations has revealed similar mechanisms whereby reductions in self-efficacy and QOL influence mental health through complex psychological processes, such pathways have not been systematically characterized in VSS populations.

Our findings corroborate and extend existing literature on mental health outcomes in VSS. Consistent with prior reports^7,8^, we observed significantly elevated depression levels and diminished QOL among VSS patients compared with healthy controls. These patterns mirror those documented across diverse chronic disease populations, where QOL impairment has been consistently identified as a robust predictor of psychological distress^30–33^. Similarly, accumulating evidence indicates that compromised self-efficacy confers heightened vulnerability to depressive symptoms and suicidal ideation^9,27,34–36^ though the specific pathways linking these constructs in VSS have remained unexplored.

The differential mediation patterns for depression versus suicidal ideation provide important insights into the mechanisms linking VSS to mental health outcomes. For depression, the significant direct effect alongside the mediation pathway suggests VSS operates through dual mechanisms: (1) abnormal thalamocortical connectivity and hyperexcitability in visual processing regions reported in neuroimaging studies^2,37,38^—which may directly contribute to mood dysregulation, and (2) indirect psychological pathways through impaired self-efficacy and QOL reflecting the cumulative burden of chronic visual disturbances on daily functioning^39^. This dual-pathway model aligns with research on other chronic conditions showing that depression can arise both from disease-specific neurobiological processes and from psychosocial consequences of chronic illness^40^. In contrast, the complete mediation pattern observed for suicidal ideation—where the direct effect was not significant—suggests that VSS influences suicidal ideation primarily through its downstream effects on self-efficacy and QOL rather than through direct disease-specific mechanisms. This finding is consistent with theoretical models positing that suicidal ideation typically develops through accumulation of psychosocial risk factors, particularly impaired QOL and loss of functional capacity, rather than being a direct consequence of specific disease pathology^41,42^. The literature on suicide risk in chronic illness supports this interpretation, demonstrating that suicidal ideation is more strongly predicted by psychosocial factors such as quality of life, social isolation, and hopelessness than by disease severity or specific symptoms^43,44^. In VSS populations, the loss of self-efficacy resulting from uncontrollable symptoms may progressively erode QOL, and this deteriorated QOL—manifesting as impaired physical functioning, psychological distress, compromised social relationships, and environmental limitations—creates the proximal risk conditions for suicidal ideation^45^. This conceptual framework has important clinical implications, suggesting that interventions targeting self-efficacy and QOL may be effective in preventing suicidal ideation in VSS patients, even if the underlying visual symptoms cannot be fully resolved^14^.

Several limitations should be noted. First, the cross-sectional design precludes causal inference regarding the directionality of relationships among variables. Second, while the sample size (*N* = 131) is adequate for rare disease research, it remains modest for complex mediation analyses. Third, the Chinese sample may limit generalizability to other cultural contexts. Fourth, self-report measures may introduce response bias. Fifth, the study does not account for the severity of VSS symptoms. Sixth, we did not assess other factors that may influence the relationship between VSS and mental health, such as employment status, income level, social support, and symptom severity. Seventh, potential selection bias should be considered. VSS participants were recruited primarily through a patient support group, which may have resulted in a sample of individuals who are more actively seeking information or are more distressed by their symptoms compared to the broader VSS population. This might lead to an overestimation of the prevalence and severity of psychological distress in our findings. Therefore, caution is warranted when generalizing these results to all individuals with VSS. Nevertheless, this recruitment strategy was necessary and efficient for accessing a rare disease population for this exploratory study. Despite these limitations, this study provides evidence for the roles of self-efficacy and QOL in VSS-related mental health outcomes. Future longitudinal studies are needed to establish temporal relationships, and randomized controlled trials might evaluate interventions targeting self-efficacy and QOL to improve mental health outcomes in VSS patients.

## Conclusions

This study provides novel evidence that VSS patients exhibit elevated depression and suicidal ideation alongside deficits in self-efficacy and QOL. Serial mediation analysis revealed that self-efficacy and QOL sequentially mediate the relationship between VSS and mental health outcomes. While the cross-sectional design precludes causal interpretation, these findings suggest that interventions targeting self-efficacy and QOL may help improve mental health outcomes in VSS patients. Future longitudinal research is needed to examine the temporal relationships and evaluate targeted interventions for this underserved population.

## Data Availability

The datasets generated and/or analyzed during the current study are not publicly available due to privacy and confidentiality concerns but are available from the corresponding author upon reasonable request.

## Abbreviations

CES-D: Center for dpidemiologic studies depression scale
GSES: General self-efficacy scale
QOL: Quality of life
BSSI: Beck scale for suicidal ideation
VSS: Visual snow syndrome
WHOQOL-BREF: World Health Organization Quality of Life Brief Version

## References

[1] Schankin, C. J., Maniyar, F. H., Digre, K. B. & Goadsby, P. J. Visual snow’ – a disorder distinct from persistent migraine aura. *Brain* **137**, 1419–1428 (2014). 24645145 10.1093/brain/awu050

[2] Lauschke, J. L., Plant, G. T. & Fraser, C. L. Visual snow: A thalamocortical dysrhythmia of the visual pathway? *J. Clin. Neurosci.* **28**, 123–127 (2016). 26791474 10.1016/j.jocn.2015.12.001

[3] Mehta, D. G., Garza, I. & Robertson, C. E. Two hundred and forty-eight cases of visual snow: A review of potential inciting events and contributing comorbidities. *Cephalalgia Int. J. Headache*. **41**, 1015–1026 (2021). 10.1177/0333102421996355 33615842

[4] Puledda, F., Schankin, C., Digre, K. & Goadsby, P. J. Visual snow syndrome: what we know so Far. *Curr. Opin. Neurol.* **31**, 52–58 (2018). 29140814 10.1097/WCO.0000000000000523

[5] Kavalidou, K., Smith, D. J. & O’Connor, R. C. The role of physical and mental health Multimorbidity in suicidal ideation. *J. Affect. Disord*. **209**, 80–85 (2017). 27888724 10.1016/j.jad.2016.11.026

[6] Waern, M., Rubenowitz, E. & Wilhelmson, K. Predictors of suicide in the old elderly. *Gerontology* **49**, 328–334 (2003). 12920354 10.1159/000071715

[7] Solly, E. J., Clough, M., Foletta, P., White, O. B. & Fielding, J. The psychiatric symptomology of visual snow syndrome. *Front. Neurol.* **12**, 703006 (2021). 34393980 10.3389/fneur.2021.703006 PMC8362098

[8] Van Dongen, R. M., Waaijer, L. C., Onderwater, G. L. J., Ferrari, M. D. & Terwindt, G. M. Treatment effects and comorbid diseases in 58 patients with visual snow. *Neurology* **93**, (2019). 10.1212/WNL.0000000000007825 PMC6669936 31213497

[9] Bandura, A. Self-efficacy: toward a unifying theory of behavioral change. *Psychol. Rev.* **84**, 191–215 (1977). 847061 10.1037//0033-295x.84.2.191

[10] Huang, Y., Li, S., Lu, X., Chen, W. & Zhang, Y. The effect of Self-Management on patients with chronic diseases: A systematic review and Meta-Analysis. *Healthc. Basel Switz.* **12**, 2151 (2024). 10.3390/healthcare12212151 PMC11544912 39517362

[11] Luo, Z. N. et al. The influence of family health on self-efficacy in patients with chronic diseases: the mediating role of perceived social support and the moderating role of health literacy. *BMC Public. Health*. **24**, 3398 (2024). 39673060 10.1186/s12889-024-20906-x PMC11639113

[12] The Whoqol Group. Development of the world health organization WHOQOL-BREF quality of life assessment. *Psychol. Med.* **28**, 551–558 (1998). 9626712 10.1017/s0033291798006667

[13] Chang, F. S. et al. Preliminary validation study of the WHO quality of life (WHOQOL) scales for people with spinal cord injury in Mainland China. *J. Spinal Cord Med.* **45**, 710–719 (2022). 33263492 10.1080/10790268.2020.1847563 PMC9542528

[14] Kerari, A., Bahari, G., Alharbi, K. & Alenazi, L. The effectiveness of the chronic disease Self-Management program in improving patients’ Self-Efficacy and Health-Related behaviors: A Quasi-Experimental study. *Healthcare* **12**, 778 (2024). 38610201 10.3390/healthcare12070778 PMC11011545

[15] Bandura, A., Freeman, W. H. & Lightsey, R. Self-Efficacy: the exercise of control. *J. Cogn. Psychother.* **13**, 158–166 (1999).

[16] Gao, Y. et al. The relationship between self-efficacy, health literacy, and quality of life in patients with chronic diseases: a cross-sectional study in China. *Front. Public. Health*. **12**, 1430202 (2024). 39391157 10.3389/fpubh.2024.1430202 PMC11466233

[17] Ren, L., Li, Z., Wu, J., Duan, L. & Gao, J. Knowledge, Attitudes, and practices among elderly CHD patients towards Self-Perceived health abilities. *J. Multidiscip Healthc.* **17**, 1999–2011 (2024). 38706499 10.2147/JMDH.S463043 PMC11070164

[18] Ki, M. et al. A systematic review of psychosocial protective factors against suicide and suicidality among older adults. *Int. Psychogeriatr.* **36**, 346–370 (2024). 38305360 10.1017/S104161022300443X

[19] Wen, Z. et al. Mediating effect of social support and resilience between loneliness and depression in older adults: A systematic review and meta-analytic structural equation modeling. *J. Affect. Disord*. **365**, 246–257 (2024). 39147150 10.1016/j.jad.2024.08.062

[20] Cudris-Torres, L. et al. Quality of life in the older adults: the protective role of self-efficacy in adequate coping in patients with chronic diseases. *Front. Psychol.* **14**, 1106563 (2023). 37089743 10.3389/fpsyg.2023.1106563 PMC10117781

[21] Melin, J., Fors, A., Jakobsson, S., Krabbe, D. & Björkman, I. Self-Efficacy to manage chronic disease (SEMCD) scale: translation and evaluation of measurement properties for a Swedish version. *Arch. Public. Health Arch. Belg. Sante Publique*. **81**, 2 (2023). 10.1186/s13690-022-01022-x PMC9814208 36600298

[22] Cheung, S. K. & Sun, S. Y. K. Assessment of optimistic Self-Beliefs: further validation of the Chinese version of the general Self-Efficacy scale. *Psychol. Rep.* **85**, 1221–1224 (1999). 10710976 10.2466/pr0.1999.85.3f.1221

[23] Cheung, Y. B., Yeo, K. K., Chong, K. J., Khoo, E. Y. H. & Wee, H. L. Measurement equivalence of the English, Chinese and Malay versions of the world health organization quality of life (WHOQOL-BREF) questionnaires. *Health Qual. Life Outcomes*. **17**, 67 (2019). 30995918 10.1186/s12955-019-1130-0 PMC6469132

[24] Niu, L. et al. Factor structure and measurement invariance of the Chinese version of the center for epidemiological studies depression (CES-D) scale among undergraduates and clinical patients. *BMC Psychiatry*. **21**, 463 (2021). 34556088 10.1186/s12888-021-03474-x PMC8459481

[25] Zhang, J. & Brown, G. K. Psychometric properties of the scale for suicide ideation in China. *Arch. Suicide Res.* **11**, 203–210 (2007). 17453698 10.1080/13811110600894652 PMC3210860

[26] De Beurs, D. P., Fokkema, M., De Groot, M. H., De Keijser, J. & Kerkhof, A. J. F. M. Longitudinal measurement invariance of the Beck scale for suicide ideation. *Psychiatry Res.* **225**, 368–373 (2015). 25571773 10.1016/j.psychres.2014.11.075

[27] Hayes, A. F. Introduction to Mediation, Moderation, and conditional process analysis. *Commun. Monogr.* **85**, 4–40 (2018).

[28] Vilagut, G., Forero, C. G., Barbaglia, G. & Alonso, J. Screening for depression in the general population with the center for epidemiologic studies depression (CES-D): A systematic review with Meta-Analysis. *PloS One*. **11**, e0155431 (2016). 27182821 10.1371/journal.pone.0155431 PMC4868329

[29] Chin, W. Y., Choi, E. P. H., Chan, K. T. Y. & Wong, C. K. H. The psychometric properties of the center for epidemiologic studies depression scale in Chinese primary care patients: factor Structure, construct Validity, Reliability, sensitivity and responsiveness. *PloS One*. **10**, e0135131 (2015). 26252739 10.1371/journal.pone.0135131 PMC4529142

[30] Chan, S. W. C. Chronic disease Management, Self-Efficacy and quality of life. *J. Nurs. Res.* **29**, e129 (2021). 33427791 10.1097/JNR.0000000000000422 PMC7808345

[31] Hohls, J. K., König, H. H., Quirke, E., Hajek, A. & Anxiety Depression and quality of Life—A systematic review of evidence from longitudinal observational studies. *Int. J. Environ. Res. Public. Health*. **18**, 12022 (2021). 34831779 10.3390/ijerph182212022 PMC8621394

[32] Mao, Z., Pepermans, K. & Beutels, P. Relating mental health, health-related quality of life and well-being in the aftermath of the COVID-19 pandemic: A cross-sectional comparison in 14 European countries in early 2023. *Public. Health*. **238**, 16–22 (2025). 39579613 10.1016/j.puhe.2024.11.010

[33] Büsselmann, M. et al. High quality of life reduces Depression, Hopelessness, and suicide ideations in patients in forensic psychiatry. *Front. Psychiatry*. **10**, 1014 (2020). 32038334 10.3389/fpsyt.2019.01014 PMC6989536

[34] Hawton, K., Van Heeringen, K. & Suicide *Lancet* **373**, 1372–1381 (2009). 19376453 10.1016/S0140-6736(09)60372-X

[35] Jannini, T. B., Mordacchini, I., Rossi, R., Socci, V. & Lorenzo, G. D. The influence of age on the relationship between future anxiety, loneliness, and quality of life: evidence from a sample of 5409 individuals in the general population in Italy. *Curr. Psychol.* **43**, 30148–30155 (2024).

[36] Pu, J., Hou, H. & Ma, R. Direct and indirect effects of Self-efficacy on depression: the mediating role of dispositional optimism. *Curr. Psychol.* **36**, 410–416 (2017).

[37] Hepschke, J. L. et al. Cortical oscillatory dysrhythmias in visual snow syndrome: a magnetoencephalography study. *Brain Commun.* **4**, fcab296 (2022). 35169699 10.1093/braincomms/fcab296 PMC8833316

[38] Klein, A. & Schankin, C. J. Visual snow syndrome as a network disorder: A systematic review. *Front. Neurol.* **12**, 724072 (2021). 34671311 10.3389/fneur.2021.724072 PMC8521005

[39] Puledda, F. et al. Insular and occipital changes in visual snow syndrome: a BOLD fMRI and MRS study. *Ann. Clin. Transl Neurol.* **7**, 296–306 (2020). 32154676 10.1002/acn3.50986 PMC7086005

[40] McLachlan, K. J. J. & Gale, C. R. The effects of psychological distress and its interaction with socioeconomic position on risk of developing four chronic diseases. *J. Psychosom. Res.* **109**, 79–85 (2018). 29680578 10.1016/j.jpsychores.2018.04.004 PMC5959313

[41] Gürhan, N., Beşer, N. G., Polat, Ü. & Koç, M. Suicide risk and depression in individuals with chronic illness. *Community Ment Health J.* **55**, 840–848 (2019). 30848413 10.1007/s10597-019-00388-7

[42] Kim, S. H. Suicidal ideation and suicide attempts in older adults: influences of chronic illness, functional limitations, and pain. *Geriatr. Nurs. N Y N*. **37**, 9–12 (2016). 10.1016/j.gerinurse.2015.07.006 26318163

[43] Goldmann, E., Roberts, E. T. & Parikh, N. S. Boden-Albala, B. Chronic physical illness burden and suicidal ideation among Dominicans in new York City. *J. Immigr. Minor. Health*. **19**, 616–622 (2017). 27507022 10.1007/s10903-016-0477-0

[44] Rahouma, M. et al. Lung cancer patients have the highest malignancy-associated suicide rate in USA: a population-based analysis. *Ecancermedicalscience* **12**, 859 (2018). 30174721 10.3332/ecancer.2018.859 PMC6113987

[45] Miskowiak, K. W. et al. Predictors of the discrepancy between objective and subjective cognition in bipolar disorder: a novel methodology. *Acta Psychiatr Scand.* **134**, 511–521 (2016). 27644707 10.1111/acps.12649

